# *Ganoderma tsugae* prevents cognitive impairment and attenuates oxidative damage in d-galactose-induced aging in the rat brain

**DOI:** 10.1371/journal.pone.0266331

**Published:** 2022-04-07

**Authors:** Hui-Chuan Kuo, Sih-Yu Tong, Ming-Wei Chao, Chia-Yi Tseng

**Affiliations:** 1 Department of Pharmacy, Taoyuan General Hospital, Ministry of Health and Welfare, Taoyuan District, Taoyuan City, Taiwan; 2 Department of Biomedical Engineering, Chung Yuan Christian University, Zhongli District, Taoyuan City, Taiwan; 3 Department of Bioscience Technology, Chung Yuan Christian University, Zhongli District, Taoyuan City, Taiwan; Zagazig University, EGYPT

## Abstract

Lingzhi has long been regarded as having life-prolonging effects. Research in recent years has also reported that Lingzhi possesses anti-tumor, anti-inflammatory, immunomodulatory, hepatoprotective, and anti-lipogenic effects. The D-galactose (D-gal, 100 mg/kg/day)-induced aging Long-Evans rats were simultaneously orally administered a DMSO extract of *Ganoderma tsugae* (GTDE, 200 μg/kg/day) for 25 weeks to investigate the effects of GTDE on oxidative stress and memory deficits in the D-galactose-induced aging rats. We found that GTDE significantly improved the locomotion and spatial memory and learning in the aging rats. GTDE alleviated the aging-induced reduction of dendritic branching in neurons of the hippocampus and cerebral cortex. Immunoblotting revealed a significant increase in the protein expression levels of the superoxide dismutase-1 (SOD-1) and catalase, and the brain-derived neurotrophic factor (BDNF) in rats that received GTDE. D-gal-induced increase in the lipid peroxidation product 4-hydroxynonenal (4-HNE) was significantly attenuated after the administration of GTDE, and pyrin domain-containing 3 protein (NLRP3) revealed a significant decrease in NLRP3 expression after GTDE administration. Lastly, GTDE significantly reduced the advanced glycosylation end products (AGEs). In conclusion, GTDE increases antioxidant capacity and BDNF expression of the brain, protects the dendritic structure of neurons, and reduces aging-induced neuronal damage, thereby attenuating cognitive impairment caused by aging.

## Introduction

Aging, a slow and progressive process of physiological change, is an inevitable natural phenomenon that is irreversible. The rate at which aging occurs is affected by complex interactions among genetics, the environment, diet, and lifestyle habits. Aging is often accompanied by a decline in physiological function, which decreases activities of daily living (ADL) and, consequently, increases healthcare and social resource demands [1, 2]. Several theories have been postulated to explain the process of aging, with the most renowned being the free radical theory of aging (FRTA) proposed by Dr. Denham Harman in 1956 [3]. According to the FRTA, aging is caused by free radicals in the body, which induce lipid peroxidation, protein oxidation, and DNA damage. Previous literature has reported that oxidative stress plays a key role in the pathogenesis of neurological or neurodegenerative diseases associated with aging [4, 5], such as Parkinson’s disease [6] and Alzheimer’s disease [7]. Studies have also shown that reactive oxygen species (ROS) promote the inflammatory response through the upregulation of pro-inflammatory mediators, which leads to the formation of inflammasomes in cells. The over-accumulation of inflammasomes in neurons may lead to neuronal death and degeneration [8–10]. Therefore, the prevention of oxidative stress has become a major direction for research on the delay of aging and the treatment of neurodegenerative diseases.

D-galactose (D-gal) is a reducing sugar that produces ROS when it is metabolized in the body [11]. It interacts with free amines on proteins and peptides via non-enzymatic glycation, producing advanced glycosylation end products (AGEs) that indirectly stimulate the production of ROS and, ultimately, cause oxidative stress [12, 13]. Research has shown that AGEs promote the formation and deposition of neurofibrillary tangles and amyloid plaques, which are neuropathological hallmarks of Alzheimer’s disease [14]. Long-term processing of D-gal causes oxidative stress [15], neuronal apoptosis [16], and inflammatory response [17], thereby accelerating aging and resulting in reduced learning ability, memory, and motor ability in animals [18, 19]. Therefore, D-gal-induced animal aging models can be used for the simulation of the characteristics of natural aging in the brain or age-associated neurodegenerative diseases. Models have been recognized internationally and widely used for research on aging mechanisms [20]. D-gal has also been adopted in the present study for the construction of an aging rat model.

Lingzhi (also known as Reishi) is an edible medicinal mushroom that has been regarded as a herb with health-promoting and life-prolonging effects in East Asian countries for several centuries [21]. It contains various bioactive compounds such as polysaccharides, triterpenes, adenosine, and small-molecule proteins. Studies have indicated that Lingzhi has various effects, including anti-tumor [22], anti-inflammatory [23], hypolipidemic [24], hypoglycemic [25], antioxidant [26], immunomodulatory [27], hepatoprotective [28] and anti-atherosclerotic [29] effects. A recent study has demonstrated the medical usage of Lingzhi in vivo and clinical studies [30]. In the present study, Songshan Lingzhi (*Ganoderma tsugae*), a type of Lingzhi widely used in Asia, was selected for the treatment of D-gal-induced aging rats. The hydroxyl radical-scavenging effect and metal ion chelating ability of *G*. *tsugae* give rise to superior antioxidant effects [31, 32]. Moreover, there is currently no research to apply it to the nervous system. In the present study, we established a D-gal-induced Long-Evans rat aging model through subcutaneous injections of D-gal (100 mg/kg/day). The rats were subsequently orally administered a DMSO extract of *Ganoderma tsugae* (Songshan Lingzhi, a common type of Lingzhi) (GTDE, 200 μg/kg/day) for 25 weeks. We aimed to investigate whether the administration of *G*. *tsugae* attenuated oxidative stress and memory deficits in D-gal-induced aging rats.

## Results

### GTDE did not affect the physiological parameters of rats

We first measured the basic physiological parameters of the various groups to evaluate the effects of GTDE on the rats. As shown in Table 1, there were no significant differences in bodyweight and brain weight among the various groups (Weight gain: *p* = 0.9357, F = 0.1389; Brain weight: *p* = 0,3334, F = 1.199). The values of the physiological parameters for the D-gal+GTDE group were similar to those for the control group. Compared with the control group, the D-gal group had a slightly lower hemoglobin concentration, slightly higher red blood cell (RBC) count, and decreased white blood cell (WBC) count (Hb: *p* = 0.8843, F = 0.2155 WBC: *p* = 0.7221, F = 0.4484; RBC, *p* = 0.5034, F = 0.8190). In aging rats fed with GTDE, a trend of recovery towards the values of the control group was observed in these parameters, but the differences in the various physiological parameters were not statistically significant. These experimental results indicate that injections of D-gal and the oral administration of GTDE for 25 weeks did not affect the basic physiological parameters of the rats.

**Table 1:** Changes in physiological parameters of Long-Evans rats after oral administration of *G*. *tsugae*.

Parameters/ Groups	Control	GTDE	D-gal	D-gal + GTDE
Weight Gain (%)	15.87 ± 3.62	15.17 ± 7.27	19.59 ± 7.18	18.47 ± 2.77
Brain Weight (g)	2.22 ± 0.04	2.26 ± 0.06	2.32 ± 0.07	2.36 ± 0.05
Hemoglobin (g/dL)	13.75 ± 0.59	13.67 ± 0.50	12.97 ± 1.05	13.67 ± 1.01
RBC (109/mL)	5.38 ± 0.38	6.69 ± 0.95	8.45 ± 1.64	8.37 ± 2.03
WBC (106/mL)	3.29 ± 0.27	3.04 ± 0.41	2.63 ± 0.29	3.43 ± 0.68

^1^ The differences in body weight, brain weight, hemoglobin level, RBC count, and WBC count were not statistically significant. The physiological parameters were compared using the one-way ANOVA followed by the Tukey’s multiple comparisons test. Means ± SEM, n = 7.

### GTDE improved alertness to the environment in d-gal-induced aging rats

**Fig 1:**
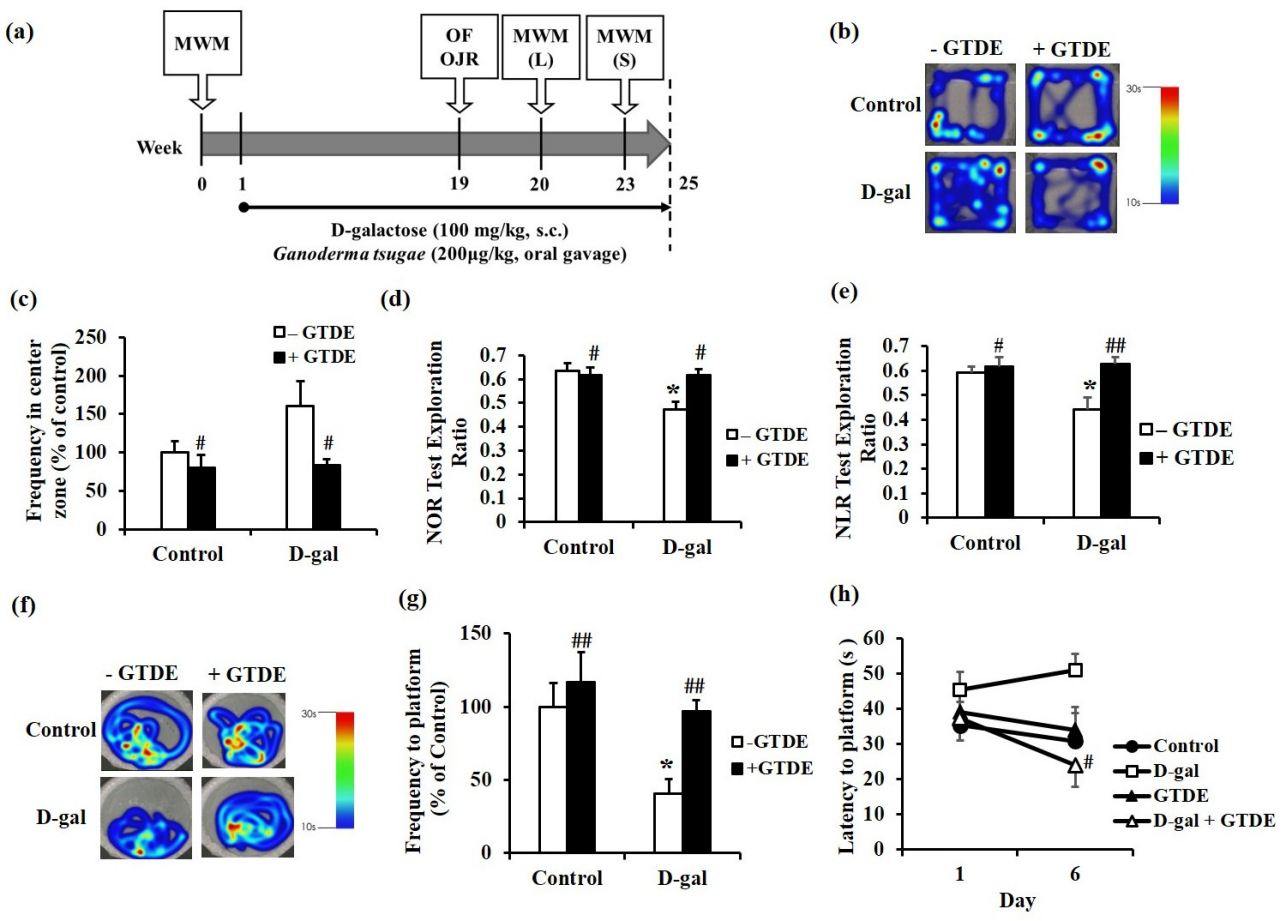
GTDE restored recognition memory against d-gal induced aging. The rats received daily drug administrations based on group assignments. D-gal was administered by subcutaneous injection at a dose of 100 mg/kg/day, and GTDE was administered orally at a dose of 200 μg/kg/day. Rats in the control group received equivalent volumes of the solvent. At 18 weeks after the commencement of drug administration, the rats were subjected to the open-field test ((b), (c)), object recognition test ((d), (e)), and Morris water maze test ((f) to (h)). **(a)** The detailed experimental schedule. (b) The heat map for the open-field test, with red denoting the maximum duration of stay of 30 s and blue denoting the minimum duration of 10 s. (c) The quantified frequencies at which the rats reached the central zone. (d) Results of the NOR test. (e) Results of the NLR test. (f) Heat map for the Morris water maze test, with red denoting the maximum duration of stay of 30 s and blue denoting the minimum duration of 10 s. (g) Quantified frequencies of platform exploration during the long-term memory test. (h) Results of the working memory test. Intergroup differences were compared using one-way ANOVA followed by the Tukey’s multiple comparisons test. Statistically significant differences based on comparisons with the control group are denoted by *P<0.05, **P<0.01, ***P<0.001; statistically significant differences based on comparisons with the D-gal group are denoted by ^#^*P*<0.05, ^##^*P*<0.01, ^###^*P*<0.001. n = 7 per group.

The open-field test is often used for the evaluation of behaviors or emotions such as locomotion, exploration, and anxiety by taking advantage of the thigmotactic behavior of animals [33, 34]. In this study, the test was performed 18 weeks after the commencement of D-gal injections and oral administration of GTDE (Fig 1a). Fig 1b shows the heat map of rat activity during the test. The colors on the heat map represent the duration spent crossing or staying in a certain zone, with red denoting the maximum duration of 30 s and blue denoting the minimum duration of 10 s. As shown in the figure, the rats in all the groups, except the D-gal group, generally exhibited thigmotactic behavior. Therefore, we further analyzed the frequencies with which the rats remained in the central zone. The results shown in Fig 1c (*p < 0*.*05* using one-way ANOVA followed by the Tukey’s multiple comparisons test, F = 3.636, *p* = 0.0328) indicate that the frequency with which the rats entered the central zone was highest in the D-gal group compared with the other groups, whereas the frequency was significantly lower in the D-gal+GTDE group than in the D-gal group.

### GTDE improved the short-term memory of d-gal-induced aging rats

Both the familiar/novel object recognition test and familiar/novel location recognition test are methods for high-level verification of recognition memory [35]. The novel object recognition (NOR) and novel location recognition (NLR) tests were carried out 18 weeks after the commencement of D-gal injections and oral administration of GTDE (Fig 1a). The results showed that the D-gal group spent a significantly shorter time exploring novel objects (Fig 1d) and novel locations (Fig 1e) than exploring familiar objects and familiar locations. This suggested the impairment of the recognition of objects and locations in the D-gal group, which demonstrates a decrease in short-term memory. The time spent exploring novel objects and novel locations increased in the D-gal+GTDE group, indicating that the administration of GTDE attenuated the deterioration of short-term memory (Fig 1d and 1e) (Fig 1d: *p < 0*.*05* using one-way ANOVA followed by the Tukey’s multiple comparisons test, F = 5.158, *p* = 0.0075; Fig 1e: *p < 0*.*05* using one-way ANOVA followed by the Tukey’s multiple comparisons test, F = 5.442, *p* = 0.0059).

### GTDE improved spatial memory impairment in d-gal-induced aging rats

The Morris water maze is used to assess spatial learning and memory in animals [36]. At the start of the experiment, the rats were subjected to initial training to enable the formation of long-term memory, which was validated at 19 weeks after the commencement of D-gal injections and oral administration of GTDE (Fig 1a). Fig 1f shows the heat map of rat activity during the test. The colors on the heat map represent the duration spent crossing or staying in a certain zone, with red denoting the maximum duration of 30 s and blue denoting the minimum duration of 10 s. Our results indicate that the rats in all the groups, except the D-gal group, remained on the platform for a significantly longer duration than on other zones of the maze. Therefore, we further analyzed the frequency at which the rats remained on the platform (Fig 1g: *p < 0*.*05* using one-way ANOVA followed by the Tukey’s multiple comparisons test, F = 4.976, *p* = 0.0034). The analysis shows that the D-gal group was less likely to reach the platform than the control group. By contrast, the likelihood of the D-gal+GTDE group reaching the platform was greater, which was similar to that of the control group. These results indicate that the aging rats suffered from age-associated spatial memory impairment, which was alleviated with the administration of GTDE.

### GTDE improved the working memory of d-gal-induced aging rats

Besides its use for the classical spatial long-term memory test, the Morris water maze can also be used to assess working memory, which represents the ability to learn new things, by moving the platform to a new location each day [37, 38]. The working memory test was performed 23 weeks after the D-gal injections and oral administration of GTDE. From Fig 1h, it can be observed that the time taken to locate the platform did not improve significantly with the number of days of the test in the D-gal group. By contrast, a trend of improvement was observed in the control and D-gal+GTDE groups; significant recovery was achieved by the D-gal+GTDE group. This indicates that working memory decreased with aging, but this decline could be prevented by GTDE (Fig 1h: *p* = 0.0893 using one-way ANOVA followed by the Tukey’s multiple comparisons test, F = 1.842).

### GTDE attenuated the aging-induced reduction of dendritic branching in the hippocampus and cerebral cortex

**Fig 2:**
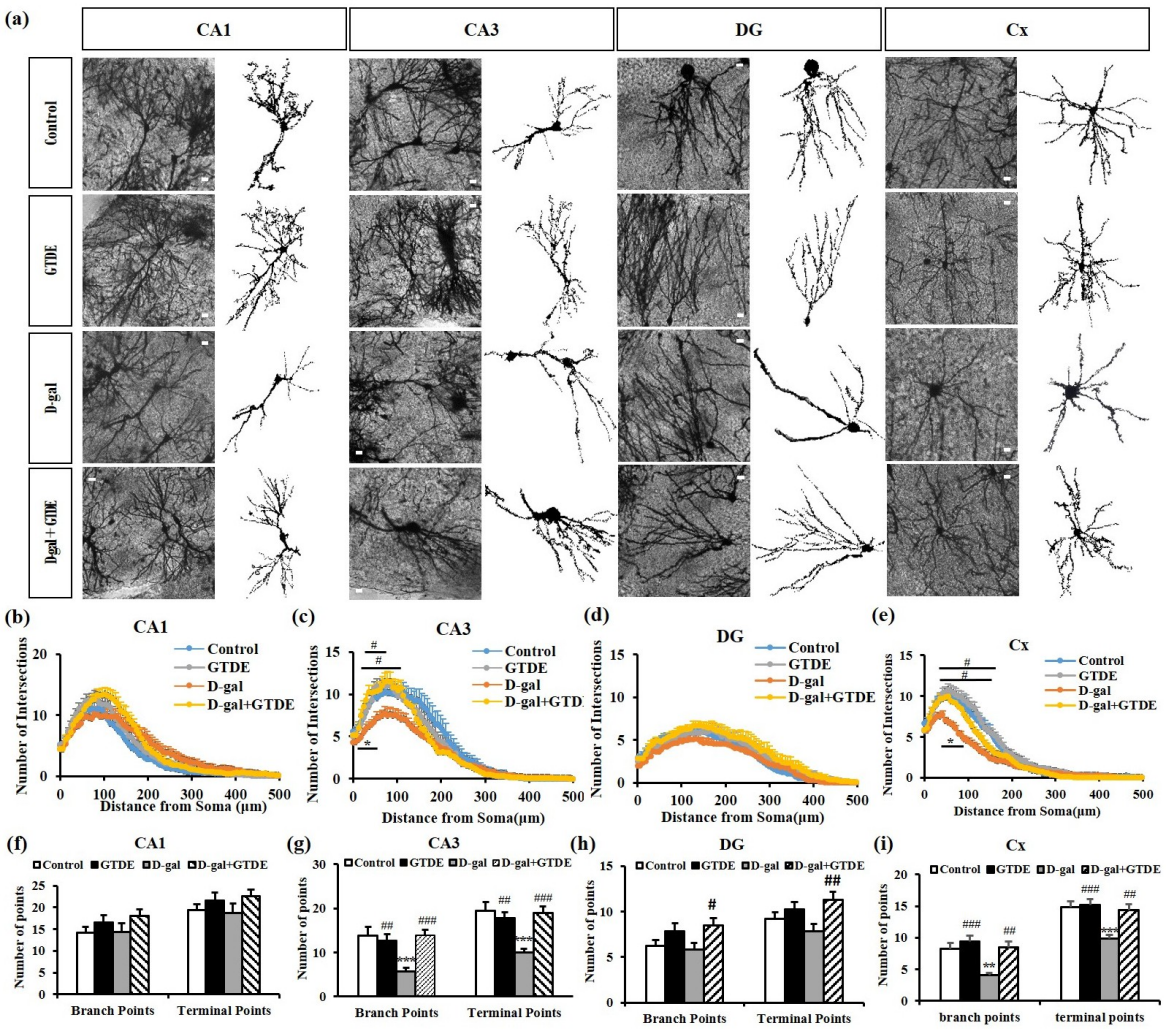
The aging-induced decrease in the number of dendritic branches in neurons of the hippocampus and cerebral cortex was attenuated by the administration of GTDE. (a) Morphology of neurons in the Golgi-stained hippocampal and cerebral cortical sections of the rats (scale bar = 50 μm). (b) to (e) The results of (a) Sholl analysis for the (b) hippocampal CA1 region, (c) hippocampal CA3 region, (d) hippocampal dentate gyrus region, and (e) cerebral cortex. (f-i) Results of the analysis of the number of dendritic branch points and terminal points: (f) hippocampal CA1 region; (g) hippocampal CA3 region; (h) hippocampal dentate gyrus region; and (i) cerebral cortex. Intergroup differences were compared using one-way ANOVA followed by the Tukey’s multiple comparisons test. Statistically significant differences based on comparisons with the control group are denoted by *P<0.*05*, **P<0.*01*, ***P<0.*001*; statistically significant differences based on comparisons with the D-gal group are denoted by ^*#*^*P<0*.*05*, ^*##*^*P<0*.*01*, ^*###*^*P<0*.*001*. n (images) value (CA1, CA3, DG, Cx) = control (21,21,22,23); GTDE(16,14,18,25); D-gal (16,16,19,23); D-gal+GTDE (18,17,16,23).

The complexity of the dendritic branching determines whether a good synaptic function exists in neurons and affects their ability to process and consolidate information [39]. Fig 2a shows the representative Golgi stains of the various groups. Fig 2b–2e show the Sholl analysis results of dendritic branching in the hippocampus and cerebral cortex, and Fig 2f–2i show the total dendritic branch points and terminal points in the hippocampus and cerebral cortex. The results indicate the following: (1) Regarding the dendritic branch intersections in the hippocampal CA1 region, the D-gal group had fewer intersections than the control group, and the D-gal+GTDE group had slightly more intersections than the D-gal group. However, the differences among the various groups were not statistically significant (Fig 2b: *p > 0*.*05* using one-way ANOVA followed by the Tukey’s multiple comparisons test, 108 μm from the soma: F = 2.367, *p* = 0.0786; 120 μm from the soma: F = 2.559, *p* = 0.0623). Fig 2f shows the number of dendritic branch points and terminal points for each group. The D-gal group did not differ from the control group based on the number of dendritic branch points, but it had slightly fewer dendritic terminal points than the control group. Both the number of dendritic branch points and the number of dendritic terminal points were slightly higher in the D-gal+GTDE group than in the control group, but the differences were not statistically significant (Fig 2f: *p > 0*.*05* using one-way ANOVA followed by the Tukey’s multiple comparisons test, branch points: F = 1.442, *p* = 2.2384; terminal points: F = 1.149, *p* = 0.3359); (2) Sholl analysis of the neurons of the hippocampal CA3 region of the various groups showed fewer intersections in the D-gal group than in the control group—the difference was statistically significant within distances of 25–50 μm from the soma (Fig 2c: *p < 0*.*05* using one-way ANOVA followed by the Tukey’s multiple comparisons test, 36 μm from the soma: F = 7.271, *p* = 0.0003; 108 μm from the soma: F = 3.671, *p* = 0.0166). They were significantly more branch points in the D-gal+GTDE and D-gal groups within distances of 20–108 μm (Fig 2c). The counts of the dendritic branch points and terminal points in Fig 2g indicate that the dendritic branch points and terminal points of the D-gal group were fewer than those of the control group, with the differences between the two groups being statistically significant. The D-gal+GTDE group had more dendritic branch points and terminal points than the D-gal group, with the differences between the two groups being statistically significant (Fig 2g: *p < 0*.*05* using one-way ANOVA followed by the Tukey’s multiple comparisons test, branch points: F = 7.933, *p* = 0.0001; terminal points: F = 9.719, *p*<0.0001); (3) Sholl analysis of the neurons of the hippocampal dentate gyrus region of the various groups showed that the D-gal group had fewer intersections than the control group, but the difference was not statistically significant (Fig 2d). Compared with the D-gal group, the D-gal+GTDE group had more intersections within the distance of 144–156 μm, with the difference being statistically significant (Fig 2d: *p < 0*.*05* using one-way ANOVA followed by the Tukey’s multiple comparisons test, 144 μm from the soma: 4.371, *p* = 0.0064; 156 μm from the soma: 4.590, *p* = 0.0049). The dendritic branch points and terminal points were fewer in the D-gal group than in the control group, but the differences were not statistically significant. The D-gal+GTDE group had more dendritic branch points and terminal points than the D-gal group; the difference in the number of dendritic terminal points was statistically significant (Fig 2h: *p < 0*.*05* using one-way ANOVA followed by the Tukey’s multiple comparisons test, branch points: F = 2.852, *p* = 0.0433; terminal points: F = 9.719, *p*<0.0001). These demonstrated that the administration of GTDE attenuated the aging-induced decrease in dendritic branching in certain hippocampal regions of aging rats.

The results of Sholl analysis of the neurons in the cerebral cortex indicate that the D-gal group had fewer intersections than the control group, with the difference being statistically different within distances of 60–162 μm (Fig 2e). Compared with the D-gal group, the D-gal+GTDE group had more intersections within the distances of 60–100 μm, with the difference being statistically significant (Fig 2e: *p < 0*.*05* using one-way ANOVA followed by the Tukey’s multiple comparisons test, 60 μm from the soma: F = 8.863, *p*<0.0001; 96 μm from the soma: F = 9.264, *p*<0.0001). From Fig 2i, the branch points and terminal points were significantly fewer in the D-gal group than in the control group. However, the D-gal+GTDE group had significantly more branch points and terminal points than the D-gal group. The D-gal+GTDE group also had significantly more branch points and terminal points than the control group, although the difference was not statistically significant (Fig 2i: *p < 0*.*05* using one-way ANOVA followed by the Tukey’s multiple comparisons test, branch points: F = 8.440, *p*<0.0001; terminal points: F = 8.599, *p*<0.0001). The results above indicate that GTDE attenuated the aging-induced decrease in dendritic branching in the cerebral cortex of aging rats.

### GTDE decreased lipid peroxidation product expression

**Fig 3:**
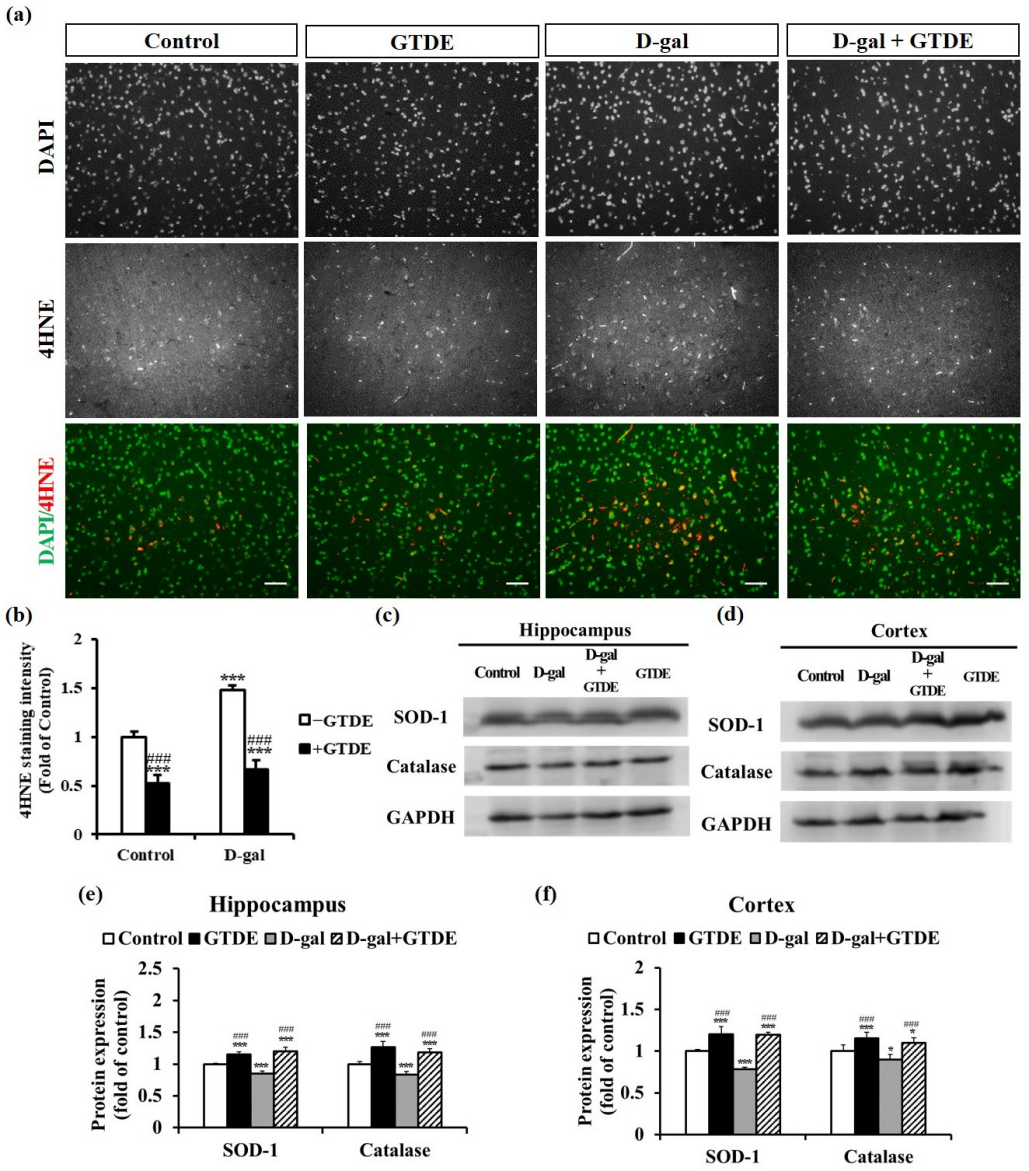
GTDE effectively reduced d-gal-induced oxidative stress and increased the expression levels of antioxidant proteins and BDNF. (a) Expression of the lipid peroxidation product 4-HNE in the cerebral cortical sections of the rats (scale bar = 50 μm). n (images) value = 21 (control); 20 (GTDE); 23 (D-gal); 21 (D-gal+aging). (b) Quantitative results of 4-HNE staining intensity. (c) to (f) Western blots of the proteins obtained from the homogenization of the hippocampal and cerebral cortical tissues of the rats. The changes in SOD-1 and catalase expressions were compared with the use of GAPDH as the loading control. n = 9. (c) and (d) Representative western blots for the hippocampus and cerebral cortex, respectively. (e) and (f) Corresponding quantified results. Intergroup differences were compared using one-way ANOVA followed by the Tukey’s multiple comparisons test. Statistically significant differences based on comparisons with the control group are denoted by *P<0.05, **P<0.01, ***P<0.001; statistically significant differences based on comparisons with the D-gal group are denoted by ^#^*P*<0.05, ^##^*P*<0.01, ^###^*P*<0.001.

To determine if GTDE alleviated the effects of aging on neuronal morphology and function through its antioxidant activity, we assessed the concentration of the lipid peroxidation product 4-hydroxynonenal (4-HNE) in the cerebral cortex. When oxidative stress causes damage in the body, 4-HNE is produced from the oxidation of polyunsaturated fatty acids, which makes it a suitable marker of oxidative damage [40]. Fig 3a and 3b show the representative immunofluorescence images and quantified results of the repeated tests, respectively. The data indicate that the 4-HNE staining intensity in the cerebral cortex was significantly higher in the D-gal group than in the control group. The administration of GTDE led to a decrease in the 4-HNE staining intensity (Fig 3a and 3b: *p < 0*.*05* using one-way ANOVA followed by the Tukey’s multiple comparisons test, F = 59.794, *p*<0.0001).

### GTDE increased antioxidant enzyme expression in the brain

The expression levels of antioxidant proteins were measured to validate the theory that GTDE attenuated the effects of aging through its antioxidant activity. Previous research has reported a decrease in the activities of antioxidant enzymes in the body during the aging process [5]. SOD-1 and catalase are two key enzymes in the antioxidant defense system of the body. To evaluate if GTDE can attenuate oxidative damage in the brain tissues of D-gal-induced aging rats, we measured the protein expression levels of SOD-1 and catalase in the cerebral cortex (Fig 3d and 3f) (Fig 3f: *p < 0*.*05* using one-way ANOVA followed by the Tukey’s multiple comparisons test, SOD: F = 58.586, *p*<0.0001; Catalase: F = 24.958, *p*<0.0001) and hippocampus (Fig 3c and 3e) (Fig 3e: *p < 0*.*05* using one-way ANOVA followed by the Tukey’s multiple comparisons test, SOD: F = 137.19, p<0.0001; Catalase: F = 88.101, p<0.0001). Fig 3c–3f show the representative Western blots and the quantified results of the repeated tests, respectively. The results indicate that D-gal lowered the SOD-1 and catalase expression levels in the hippocampus and cerebral cortex of rats, whereas the administration of GTDE caused a reversion of the expression levels to almost normal, with the differences between the D-gal group and D-gal+GTDE group being statistically significant.

### GTDE decreased aging-induced inflammasome expression

**Fig 4:**
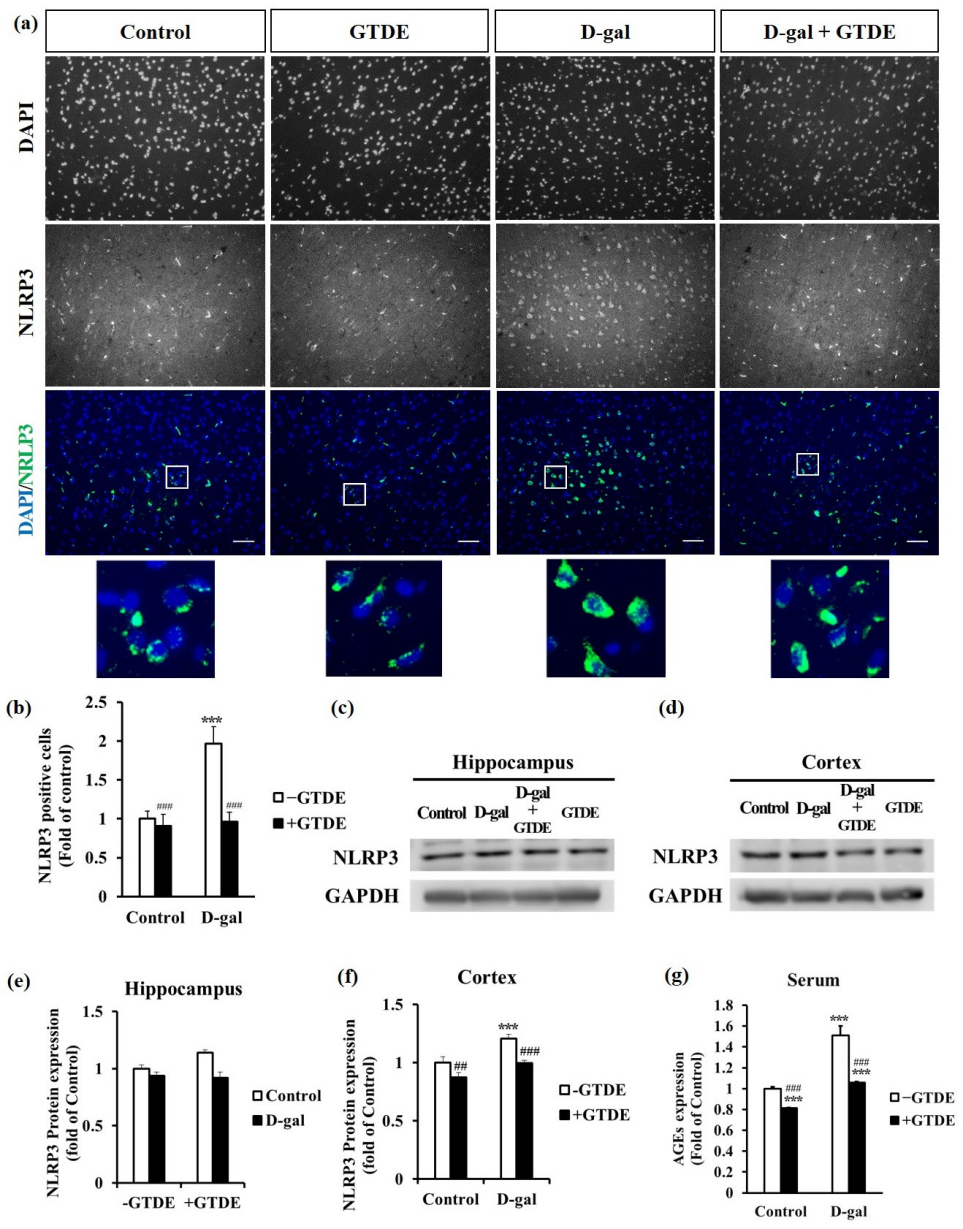
GTDE decreased the expression of the NLRP3 inflammasome and AGEs in the cerebral cortex. (a) Immunohistochemical stains for NLRP3 inflammasome expression in the cerebral cortex (scale bar = 50 μm). The squared area in the DAPI/NRLP3 merged image was enlarged and showed underneath the original image. (b) Quantified results of immunohistochemical staining, which indicates the NLRP3 positive cells. n (images) value = 9 (control); 10 (GTDE); 11 (D-gal); 11 (D-gal+aging). (c)–(f) Changes in NLRP3 expression in the hippocampus and cerebral cortex. (c)–(d) Representative Western blots for the hippocampus and cerebral cortex. (e)–(f) Corresponding quantified results. n = 6. (g) Results of the analysis of the concentration of the AGEs in serum (n = 7). Intergroup differences were compared using one-way ANOVA followed by the Tukey’s multiple comparisons test. Statistically significant differences based on comparisons with the control group are denoted by *P<0.05, **P<0.01, ***P<0.001; statistically significant differences based on comparisons with the D-gal group are denoted by ^#^*P*<0.05, ^##^*P*<0.01, ^###^*P*<0.001.

Several studies have shown that aging induces oxidative stress and causes an increased inflammatory response [41–43]. Therefore, we aimed to determine whether GTDE decreased the expression of the NLRP3 inflammasome. First, changes in the inflammasome expression in the cerebral cortex were observed by immunofluorescence. Fig 4a and 4b show the representative immunofluorescence images and the quantified NLRP3 positive cells, respectively. It can be observed that the NLRP3 positive cells in the cerebral cortex of the D-gal group were increased by 0.8 times that in the control group, whereas the NLRP3 positive cells of the D-gal+GTDE group was decreased by 0.9 times that in the D-gal group. Rats treated with GTDE only (GTDE group) also showed a minimal decrease in inflammasome expression compared with the control group (Fig 4a and 4b) (Fig 4b: *p < 0*.*05* using one-way ANOVA followed by the Tukey’s multiple comparisons test, F = 26.145, *p*<0.0001). Moreover, we found that the NLRP3 expression inside each cell increased in the D-gal induced group compared to control, which could be attenuated by GTDE administration (Fig 4a, enlarged images). Western blotting was used to measure NLRP3 protein expression in the hippocampal and cerebral cortical tissues of the rats. Fig 4c–4f show the representative Western blots and quantified results of the repeated tests, respectively. NLRP3 protein expression in the hippocampal tissue of the D-gal group was increased by 0.14 times that in the control group. By contrast, the expression level of the D-gal+GTDE group was decreased by 0.23 times that of the D-gal group, with the difference being statistically significant (Fig 4e: *p < 0*.*05* using one-way ANOVA followed by the Tukey’s multiple comparisons test, F = 30.163, *p*<0.0001). The NLRP3 protein expression level in the cerebral cortical tissue of the D-gal group was 0.2 times higher than that of the control group whereas the expression level of the D-gal+GTDE group was 0.21 times lower than that of the D-gal group, with both differences in expression level being statistically significant (Fig 4f: *p < 0*.*05* using one-way ANOVA followed by the Tukey’s multiple comparisons test, F = 17.956, *p*<0.0001). The NLRP3 protein expression levels in both the hippocampus and cerebral cortex of the GTDE group were lower than those of the control group, but the differences were not statistically significant.

### GTDE decreased formation of AGEs

Researchers have reported that aging usually causes an increase in the formation of AGEs [44, 45]. AGEs alter the structures and functions of cells by binding to receptors on cell surfaces, thereby promoting an increase in oxidative stress and the inflammatory response [46]. The experimental results described above have confirmed that both oxidative stress and the inflammatory response increased significantly in the D-gal group. Therefore, we aimed to investigate whether the formation of AGEs significantly increased in the D-gal-induced aging group and whether GTDE attenuated the accumulation of AGEs. From the results shown in Fig 4g, the concentration of the AGEs in the serum of the D-gal group was 0.51 times higher than that of the control group, whereas the concentration of the AGEs of the D-gal+GTDE group was 0.46 times lower than that of the D-gal group and comparable to that of the control group. The concentration of the AGEs in the GTDE group was 0.19 times lower than that in the control group, which further demonstrates the effects of GTDE in attenuating the formation of AGEs (Fig 4g: *p < 0*.*05* using one-way ANOVA followed by the Tukey’s multiple comparisons test, F = 39.530, *p*<0.0001).

### GTDE increased BDNF expression

**Fig 5:**
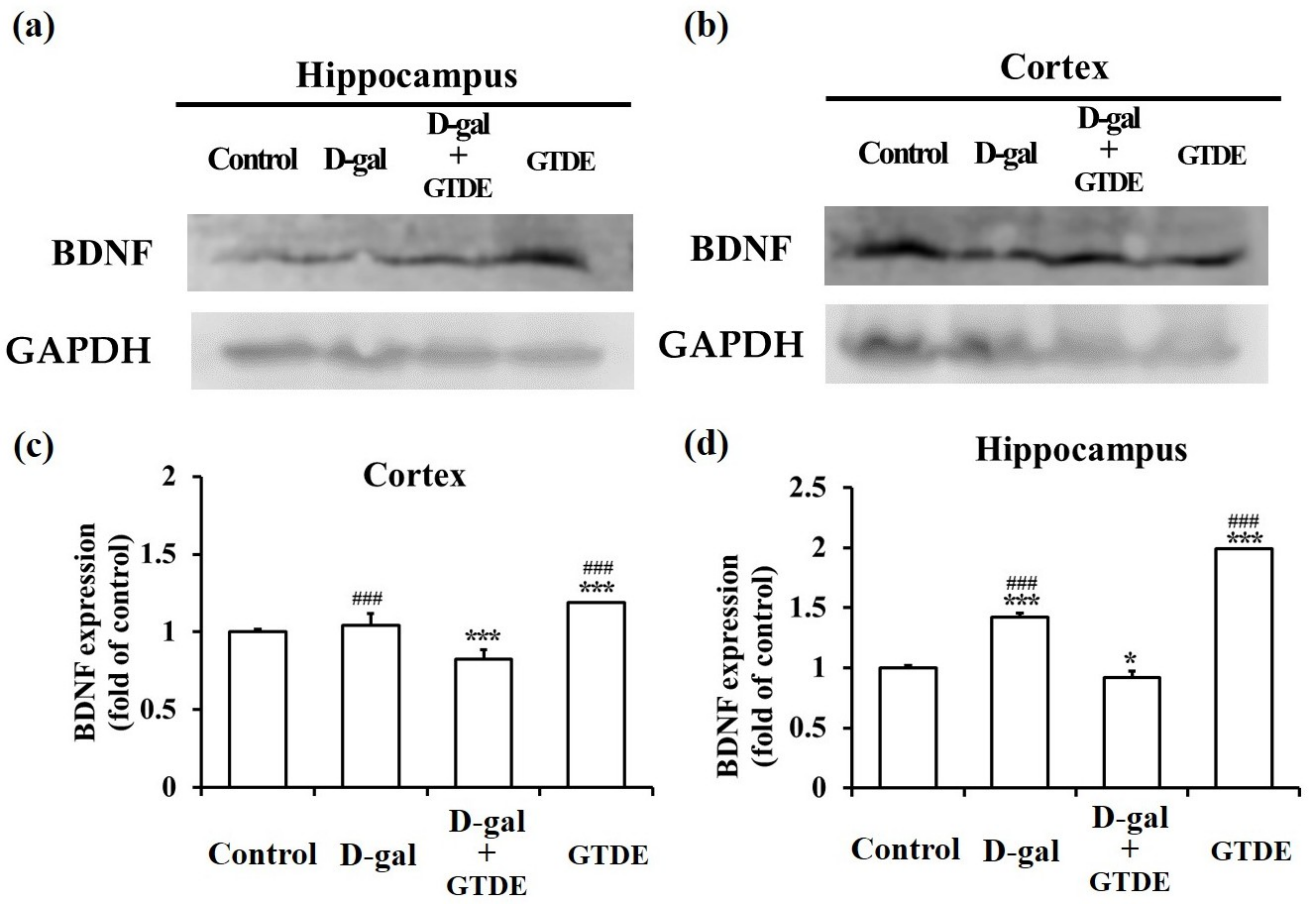
The application of GTDE efficiently increased the D-gal induced decline in the expression levels of BDNE. (a) to (d) Western blots of the proteins obtained from the homogenization of the hippocampal and cerebral cortical tissues of the rats. The change in BDNF was compared with the use of GAPDH as the loading control. (a) and (b) Representative western blots for the hippocampus and cerebral cortex, respectively. (c) and (d) Corresponding quantified results. Intergroup differences were compared using one-way ANOVA followed by the Tukey’s multiple comparisons test. Statistically significant differences based on comparisons with the control group are denoted by *P<0.05, **P<0.01, ***P<0.001; statistically significant differences based on comparisons with the D-gal group are denoted by ^#^*P*<0.05, ^##^*P*<0.01, ^###^*P*<0.001. n = 9.

BDNF promotes neuron survival and differentiation and induces synaptic plasticity. In aging brains, BDNF expression generally demonstrates a decreasing trend, which is indicative of a reduction in neurogenesis and neuronal activity [47, 48]. We aimed to determine whether GTDE was also capable of maintaining brain health by maintaining or promoting BDNF expression in addition to exerting anti-inflammatory effects. When BDNF expression levels in the hippocampal and cerebral cortex tissues of the various groups were compared by Western blotting, we found that the D-gal group had lower BDNF expression levels in both the hippocampus and cerebral cortex than in the control group, whereas the D-gal+GTDE group had higher BDNF expression levels in both types of tissues than in the D-gal group. In addition, the GTDE group also showed higher BDNF expression in both types of tissues than the control group (Fig 5a–5d: *p* < 0.05 using one-way ANOVA followed by the Tukey’s multiple comparisons tests, Cortex: F = 39.069, *p*<0.0001; Hippocampus: F = 604.00, *p*<0.0001). These results demonstrate that GTDE may promote or maintain BDNF expression in the hippocampus and cerebral cortex, which is beneficial to the maintenance of neuronal health.

## Discussion

Red reishi (*Ganoderma lucidum*) is a common species of the traditional Chinese herb, Lingzhi, and it is also commonly used in research. *G*. *lucidum* has mainly been used in clinical studies for cancer treatment [49, 50] and immunomodulation [51, 52]. Regarding cognitive function, which has been our key focus in this study, significant improvements have not yet been demonstrated in clinical research [53, 54], and evidence from animal studies remains inadequate. The present study was conducted using *Ganoderma tsugae*, another species of Lingzhi, which has better antioxidant effects than *G*. *lucidum* [31, 32]. However, there is also a lack of clinical and animal evidence for its effects on cognitive function in aging individuals. Therefore, this study is the first to demonstrate that *G*. *tsugae* attenuates aging-induced cognitive decline and protects neuronal morphology against aging through mechanisms such as antioxidant activity, reduction of inflammation, and anti-aging effects. As the dose of *G*. *tsugae* used in this study is 1/1000 of the no-observed-adverse-effect level [55], the physiological parameters of the rats that were administered GTDE did not show any significant changes (Table 1). The protective effects on cognitive function at such a low concentration provide the first animal evidence of the protection of neurocognitive function by *G*. *tsugae* (Fig 1).

The cerebral cortex and hippocampus are important brain regions that participate in key functions such as cognition, learning, and memory. Previous research has reported that dendritic structures are highly dynamic, with the number of dendritic branches decreasing with aging or the occurrence of diseases such as dementia. As dendrites are responsible for receiving signals produced by other cells, the complexity of dendritic branching determines whether a good synaptic function exists in neurons and affects the ability of neurons to process and consolidate information [56, 57]. DG, CA1, and CA3 are connected to form a typical “trisynaptic loop.” CA1 enables the cortex to recognize the signals of the hippocampal output but also can compare sensory reality with stored memory. If not, start learning. Damage of CA1 impairs long-term memory. The CA3 region is the most critical area of memory storage. CA3 damage affects long-term and short-term memory [58, 59]. Disruption dendrite patterning that sequentially affects communication between neurons leads to disruption of neuronal circuitry and finally breaks down the whole nervous system. Furthermore, many neurodegenerative diseases are defects with anomalies in dendritic morphology, such as Alzheimer’s disease and Parkinson’s disease [39]. Accordingly, the integrity of dendrite arbors is essential for maintaining the normal function of brain circuitry and neuronal networks. The present study indicates that GTDE had protective effects on cognitive behavior and was also influential in preserving neuronal dendritic morphology (Fig 2).

Such protective effects may be attributed to the superior antioxidant effects of *G*. *tsugae* to those of the other Lingzhi species [31, 32]. We found that GTDE attenuated the D-gal-induced significant increase in the concentration of the AGEs and strengthened the body’s antioxidant capacity (Fig 3). Therefore, GTDE retarded D-gal-induced aging by alleviating oxidative damage. The present study is the first evidence that illustrated the antioxidant role of G. tsugae for the nervous system. One of the typical Lingzhi species, Ganoderma Lucidum, its polysaccharide fraction, peptide, triterpenes, and ganoderic acid, has increased the SOD catalase, heme oxygenase-1, and glutathione productions and decrease the malonaldehyde expression to prevent aging [60]. This antioxidant activity is associated with the Nrf2 pathway [67] or, more specifically, Nrf2/NF-κB/NLRP3/IL-1β pathway [61].

Previous studies have stated that neuritis is the key risk factor for Parkinson’s disease and Alzheimer’s disease, and a positive correlation exists between brain inflammation and normal aging [62]. Furthermore, the over-activation of NLRP3 has been associated with several chronic diseases such as Alzheimer’s disease, diabetes mellitus, atherosclerosis, and arthritis [63]. Our findings also demonstrate that GTDE is capable of reducing aging-induced inflammatory response (Fig 4). This anti-inflammation effect might be due to inhibition of the NF-κB pathway based on the previous finding of other types of Linzhi [60, 61, 64].

We also determined if GTDE provided neuroprotective effects via other mechanisms besides attenuating oxidative stress and inflammatory response caused by aging. Therefore, we measured the levels of expression of BDNF, which modulates neurotransmitters and serves an important regulatory role in learning and memory. Previous research has reported that BDNF expression decreases gradually with increasing age [65]. Our results indicate that the administration of GTDE increased BDNF expression in the hippocampus and cerebral cortex. It can therefore be deduced that *G*. *tsugae* provides protective effects against aging-induced memory impairment by promoting BDNF expression. Furthermore, a recent study has shown that G. *lucidum* restores the BDNF level and opposes oxidative stress by stimulating the CREB/BDNF pathway via ERK1/ERK2 induction [66].

There was an unusual finding during the open-field test of this study. The open-field test assesses thigmotaxis in rodents, which is often used to evaluate behaviors or emotions such as locomotion, exploration, and anxiety [33, 34]. Fig 1 shows the significant differences between the D-gal and D-gal+GTDE groups. Therefore, the injection of D-gal may lead to abnormalities in emotions and behaviors, whereas the administration of GTDE improves behavioral disorders. The D-gal group spent more time in the central zone than the other groups; the difference between the times spent by the D-gal and D-gal+GTDE groups was significant. This shows that the aging rats had a decreased level of thigmotaxis and nervousness, leading to behaviors consistent with those observed with the administration of anxiolytic compounds in other studies [67, 68]. Such a result is inconsistent with certain literatures, which have reported that aging causes increased anxiety [69] or decreased anxiety or insignificant differences with younger animals [70]. However, our results are in agreement with those of a study by Torras-Garcia et al., which stated that the level of anxiety of rats decreased with an increase in age [71]. The differences among these studies may be attributed to differences in factors such as rearing conditions, lighting conditions, test timing, and test duration. Nonetheless, our results demonstrate that the level of thigmotaxis in rodents is maintained with the administration of GTDE.

This study had some acknowledged limitations. First, the current study is preliminary in vivo results that will need further testing in clinical to understand GTDE therapeutic potential, though other medical mushrooms have been proved to apply in clinical [30]; Second, there is no standardized method in cultivation, harvest process, and preparation of Lingzhi [72], like many other medicinal plants [73]. Therefore, the amount of Lingzhi major components yielded from extraction, such as polysaccharides and triterpenes, may vary depending on many factors, including the growing time of Lingzhi, the environment that Lingzhi is planted, the part that is used, and the solvent or method to extracting Lingzhi. The drug used in clinical must have a standard quantity of each content. Thus, an efficient criterion is needed to develop to monitor and purify the components of Lingzhi. Third, small sample sizes hide some of the relevant findings. Nevertheless, the present study is the first to demonstrate that *G*. *tsugae* significantly attenuates D-gal-induced cognitive impairment and pathological neural damage; increases the activities of SOD-1, catalase, and BDNF; decreases the concentration of AGEs and the expression of inflammatory cytokines and 4-HNE; promotes dendritic branching. Therefore, *G*. *tsugae* enhances spontaneous behavior, cognitive performance, and the antioxidant and anti-inflammatory abilities of the brain and, consequently, facilitates the effective prevention of aging-induced cognitive impairment and neurodegenerative diseases. With global aging, conditions caused by aging, including cognitive decline, which has been the focus of this study, have greatly affected the quality of life and health of older adults. Several researchers have searched for methods to alleviate or prevent the occurrence of such degenerative diseases to reduce the healthcare and long-term care burden of families and societies. Lingzhi plays a key role in nutraceuticals, which represent an emerging trend in the field of health maintenance. In future research, we hope to expand its applications to clinical studies. In that case, further evaluation is needed, such as re-validation on animal models that are genuinely aging rather than drug-induced. It is also required to find the best dose to have the significant effects in anti-oxidant, anti-inflammatory, and cognitive function improvement before used it clinically. This will, ultimately, help improve the quality of life of older adults.

## Materials and methods

### *G*. *tsugae* extraction

The *G*. *tsugae* was a gift from Professor Ruey-Shyang Hseu, Department of Biochemical Science and Technology, National Taiwan University. Dr. Hseu entrusted the Li-Kang Biotechnical Co., Ltd (I-Lan, Taiwan) to culture and collected the mycelium. He further extracted the *G*. *tsugae* from the fruiting body using hot water as described in the previously published work [74]. In brief, the fruiting body was homogenized within the sterilized water. The collected sample was frozen at -20°C vacuum-dried for 36 hr and then stored at -20°C until use. Dr. Hseu provided and authorized us to use the water extract for this study. The crude mixture contained 1.96% triterpenes and 3.93% polysaccharides [75]. The dried extract was further analyzed for the contents of total water-soluble polysaccharides, total triterpenoids, beta-D-glucans, and heavy metals. For total water-soluble polysaccharides, the sample was extracted, precipitated polysaccharides with ethanol, re-dissolved, reacted with phenol-sulfuric acid, and analyzed by UV-Vis Spectrophotometer (UV-VIS). The amount of total water-soluble polysaccharides is 45.4g/100g. For total triterpenoids, Sample was extracted and then reacted with vanillin—glacial acetic acid—perchloric acid solution as a color developing agent, analyzed by UV-Vis Spectrophotometer (UV-VIS). The amount of total triterpenoids is 463 mg/100g. For beta-D-glucans, the sample was dissolved for removing low-molecular-weight sugar, hydrolyzed by enzyme specificity, and analyzed by High Performance Anion Exchange Chromatography-Pulsed Amperometric Detector (HPAEC-PAD). The amount of beta-D-glucans is 0.24g/100g. There is no heavy metal detected. The water extract of *G*. *tsugae* was followed by DMSO precipitation, reserve dialysis, and protein depletion. The final content of triterpenoids is 128 g/100g, and the content of water-soluble polysaccharides is 0.39 g/100g in *G*. *tsugae*—DMSO solution. GTDE dosage (200 μg/kg/day) is followed by our previous preliminary study [76].

### Animals and treatments

Male Long-Evans rats (LE rats) (680–730 g) that were 24 weeks old (National Laboratory Animal Center) were used. The Institutional Animal Care and Use Committee of the Chung Yuan Christian University approved the use of animals in this study (Approved protocol no# 103019). They were kept under the following conditions: temperature of 18–26°C, the humidity of 30–70%, and a 12:12 h light-dark cycle with free access to laboratory chow and water throughout the study. The twenty-eight rats were randomly divided into four groups: (Control, GTDE treatment, D-gal treatment, and D-gal plus GTDE treatment) (n = 7 each group). In the D-gal treatment group, D-gal (100 mg/kg in PBS) was injected subcutaneously, and phosphate-buffered saline (PBS) in the same volume was given orally daily into the LE rats for 25 weeks. In the D-gal plus GTDE treatment group, GTDE (200 μg/ kg in PBS, contained 0.2% DMSO) was administered as oral gavage daily concomitantly with D-gal injections for 25 weeks. In the GTDE treatment group, oral gavage of GTDE and subcutaneous injection of PBS in the same volume was performed daily for 25 weeks. All control animals were administered PBS in the same volume subcutaneously and orally. The dosage for D-gal and GTDE references the previous literature [43] and our pilot study [76]. The schedule of the experiment’s time for 25 weeks (Fig 1a) is the reference to the previous literature [77]. The rats were sacrificed with carbon dioxide inhalation in the original feeding box, which was placed in the euthanasia container. Carbon dioxide was gradually filled the euthanasia container at a rate of 20–30% of the chamber volume per minute, causing the animal to lose consciousness quickly. Carbon dioxide was continuously perfused for another five minutes after the animal appeared dead. If rats showed anxiety during the sacrifice, 4–5% isoflurane was given to induce an anesthetic effect that caused them to lose consciousness before the carbon dioxide perfusion. Rat’s death was confirmed before removing it from the euthanasia container.

### Open-field test

An acrylic box measuring approximately 100×100×30 cm was divided by gridlines into nine equally sized squares that served as nine zones. Each rat was placed individually in the central square. Crossing was defined as the exiting of the rat from the previous square with all four paws. Between tests, the acrylic box was wiped with 75% alcohol to avoid odor interference. The frequency of crossing and duration spent in the center square within five minutes was recorded for each rat. Behavioral data were acquired by video recording and software analysis (Noldus Information Tech, Ethovision XT 10.0, Wageningen).

### Novel object recognition (NOR) test and novel location recognition (NLR) test

NOR and NLR tests were used to assess the spatial hippocampal-dependent memory of the rats. The experiment was performed based on previously described methods [78, 79]: (1) NOR test: On Days 1 and 2, the rat was placed in an acrylic box measuring 100×100×50 cm for the exploration of the same two objects for five minutes. On Day 3, one of the objects was replaced with a novel object. The duration spent exploring each object was recorded, and the preference for the novel object (%) was calculated as follows: Preference for novel object (%) = Duration spent exploring the novel object/Total duration spent exploring the two objects; (2) NLR test: On Days 4 and 5, the experiment performed on Days 1 and 2 was repeated; the rat was allowed to explore the same two objects at the same locations for five minutes. On Day 6, one of the objects was placed in a novel location. The duration spent exploring each object was recorded, and the preference (%) for the object at the novel location was calculated as follows: Preference of novel location (%) = Duration spent exploring the object at the novel location/Total duration spent exploring the two objects. Between the tests, the acrylic box was wiped with 75% alcohol to avoid odor interference.

### Morris water maze

Morris water maze has been used to evaluate sensorimotor and memory deficits in several publications [80, 81]. The rats were placed in a white circular pool with a diameter of 1.6 m positioned in a well-lit room that was painted flat black. Water was filled until 8 cm below the top of the tank, and the temperature was maintained at 25 ± 2°C. A platform (4.5 cm in diameter, 14.5 cm in height) was submerged 5 cm below the water surface and placed at the midpoint of one quadrant. Each rat underwent training for four periods per day for four consecutive days. On day five, the probe test was performed by removing the platform and allowing each rat to swim freely for 60 s. For probe trials during which the platform was removed, a differential quadrant search time and platform crossings were recorded. Behavioral data were acquired by video recording and software analysis (Noldus Information Tech, Ethovision XT 10.0, Wageningen). The video tracking software was used for analyses of escape latency and the platform frequency in the platform quadrant at the probe trial.

### Working memory test

Working memory refers to the part of short-term memory that involves storing and manipulating information for a relatively short period [82]. The experiment was carried out over six days. Each rat was placed individually in a water maze with a diameter of 160 cm and depth of 40 cm and subjected to two tests in a day. During the first test, the rat was given one minute to explore the maze and locate the submerged platform, with guidance provided if the rat failed to locate the platform. During the second test, the time taken for the rat to locate the platform within 60 seconds was recorded. The tests were repeated over the next few days; the location of the platform was changed every day and the time taken for the rat to locate the platform was recorded for each test. Behavioral data were acquired by video recording and software analysis (Noldus Information Tech, Ethovision XT 10.0, Wageningen).

### Western blotting

Brains were removed and dissected in PBS to obtain the hippocampus and cortex. The tissues were homogenized and lysed. Bradford assays were used to determine the protein concentrations of the extracts. The lysates were prepared in SDS containing sample buffer. Equal volumes with the same concentration of the lysates were separated by 10% or 12% SDS-PAGE gel using 50 μg protein per lane. The proteins were transferred to PVDF membranes using the electrophoretic transfer (Bio-Rad, Richmond, USA). The nonspecific reactivity of the membranes was blocked. The primary antibodies were rabbit anti-SOD-1 (1:1000, Abcam), rabbit anti-catalase (1:2500, Abcam), rabbit anti-BDNF (1:2000, Abcam), rabbit anti-NLRP3 (1:400, Novus Biologicals), and anti-GAPDH (Glyceraldehyde 3-phosphate dehydrogenase) (1:5,000, Sigma-Aldrich). The secondary antibody was diluted at 1:5000 in Tris-buffered saline with 0.1% Tween^®^ 20 detergent (TBST). The blots were visualized by enhanced chemiluminescence (Promega^™^ ECL Western Blotting Substrate) and analyzed on the Odyssey Infrared imaging system (LI-COR Biosciences) (Lincoln, NE). The total intensity of bands was analyzed by ImageJ (version 1.51, National Institutes of Health, USA. https://imagej.nih.gov/ij/index.html). Briefly, an area with the same size as the selected region close to the bands was used to account for background intensity. The difference between the background and the band’s integrated density was determined to be the absolute intensity of the band. Equal protein loading was confirmed using GAPDH immunoreactivity. Quantification of protein expression was normalized and presented as a fold of the control. The lysate of each brain was randomly selected. Individual homogenates were run repeated at least three times. The experimenter was blinded to the condition.

### Immunohistochemical staining

After heart perfusion, the brain tissue of each rat was removed, immersed in 4% paraformaldehyde for 24 h, allowed to sink in 30% sucrose/PBS, embedded using the optimal cutting temperature (OCT) compound (Leica, Germany), frozen in liquid nitrogen, and sectioned into 10 μm slices. The samples were air-dried overnight, the nonspecific signals were blocked, and rabbit anti-4-HNE (1:500, Novus Biologicals) and rabbit anti-NLRP3 (1:500, Novus Biologicals) were added. Subsequently, the sections were incubated with Alexa Fluro^®^ 594-conjugated AffiniPure Goat Anti-Mouse IgG and Alexa Fluor^®^ 488-conjugated AffiniPure Goat Anti-Rabbit IgG (1:250, Jackson ImmunoResearch), mounted using a DAPI (4’,6-diamidino-2-phenylindole) -containing mounting medium, and observed under a fluorescent microscope (Olympus IX51, Japan) at 200x magnification. For comparing groups, the same setting was applied in all immunostaining images, including the brightness of the excitation light and the camera exposure time. The experimenter was blinded to the condition when taking images and analyzing. Images were processed and analyzed using ImageJ. For 4-HNE, the images were thresholded with the ROI below 80 and over 200. The intensity of each image was divided by the number of DAPI positive cells. The value of each group was then normalized to control. For NLRP3, the NLRP staining positive cells were calculated and divided by the number of DAPI positive cells. The value of each group was then normalized to control.

### Measurement of the concentrations of AGEs in serum

The concentrations of AGEs were measured using the commercially available MyBioSource AGEs ELISA Kit following the manufacturer’s instructions. First, serum or plasma was diluted 20-fold using a sample diluent. The first antibody to detect AGEs is conjugated with biotin, and the second antibody is HRP-avidin. TMB (3, 3’, 5, 5’-tetramethylbenzidine) substrate was added to each well for reaction away from light at 37°C for 15–30 min. The reaction in each well was stopped by adding 50 μl of a stop solution followed by gentle plate shaking, and a color change from blue to yellow was observed after the reaction with the TMB Substrate. Absorbance at 490 nm was measured using a GloMax^®^-Multi+ Microplate Reader.

### Golgi staining procedure

Golgi staining was performed using the commercially available FD Rapid GolgiStain^™^ Kit according to the manufacturer’s instructions. Briefly, all the experimental steps were performed away from light. Approximately 1 cm^3^ of brain tissue was stained with the Golgi solutions, then embedded in a tissue freezing medium (Leica) and frozen with liquid nitrogen. The tissue was sectioned into 80 μm slices using a microtome at an operating temperature of -25°C. After being wet twice (4 min each) with deionized distilled water, the brain tissue was sequentially immersed in 50%, 75%, and 95% ethanol for 4 min each, four times in 100% ethanol (4 min each), and twice in xylene (2 min each). Lastly, the tissue sample was mounted with a mounting medium, and the CA1 region, CA3 region, and dentate gyrus of the hippocampus were observed under an optical microscope at 400x magnification. The experimenter was blinded to the condition when taking images.

### Sholl analysis

Sholl analysis was performed using the Bonfire program, a semi-automated tool for the analysis of dendrite morphology [83, 84]. The Bonfire program requires the use of two open-source analysis tools, the NeuronJ plugin for ImageJ and NeuronStudio. First, the overall morphology of the neurons was traced and recorded using ImageJ. The connectivity between the axons and dendrites was defined using NeuroStudio. Specificity analysis of the dendrite morphology was performed after the manual removal of axon tracings, and the results of the analysis were converted using the Bonfire program to an appropriate format for the removal of erroneous morphology tracing records obtained during semi-automatic tracing. Finally, the results of the Sholl and terminal/branching point analyses were simultaneously obtained. The experimenter was blinded to the condition when analyzing the dendrite morphology.

#### Statistical analysis

Data are presented as the mean ± standard error. Statistical significance (*P* < 0.05) was determined using one-way analysis of variance followed by post hoc Tukey’s Multiple Comparisons Test for multiple comparisons using GraphPad InStat software 3.05 package for Windows (GraphPad Software Inc., San Diego, CA). F- and p- values were included.

## Supporting information

S1 File (PDF)

S1 Data (XLSX)
