# Supplementary material for: Ganoderma tsugae prevents cognitive impairment and attenuates oxidative damage in d-galactose-induced aging in the rat brain
Source: PLoS One. 2022 Apr 7;17(4):e0266331. doi: 10.1371/journal.pone.0266331 (PMC8989198; doi:10.1371/journal.pone.0266331)
Supplement: S1 File — (PDF) [file pone.0266331.s001.pdf]

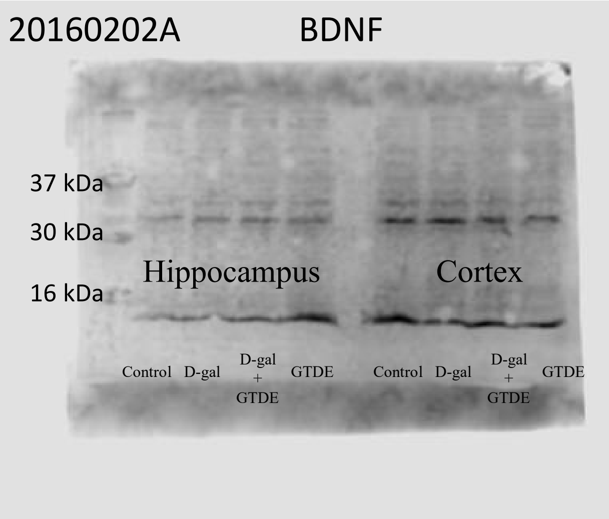
20160202A
BDNF
37 kDa
30 kDa
16 kDa
Hippocampus
Cortex
Control D-gal D-gal + GTDE GTDE Control D-gal D-gal + GTDE GTDE

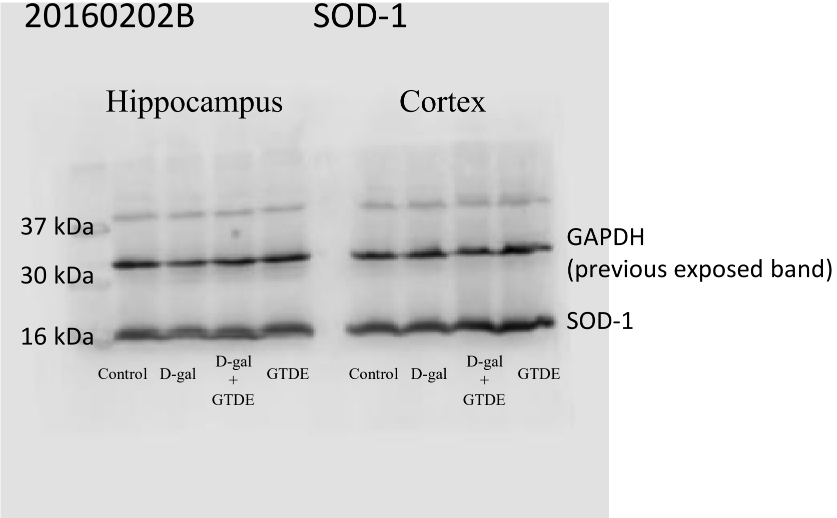
20160202B
SOD-1
Hippocampus
Cortex
37 kDa
30 kDa
16 kDa
GAPDH
(previous exposed band)
SOD-1
Control D-gal D-gal + GTDE GTDE Control D-gal D-gal + GTDE GTDE

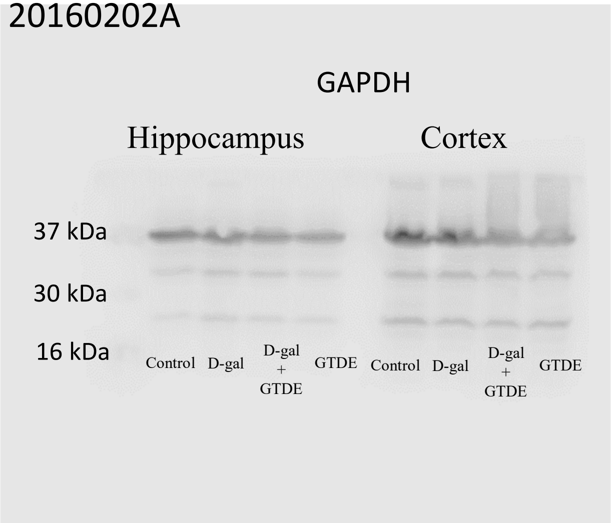
20160202A
GAPDH
Hippocampus
Cortex
37 kDa
30 kDa
16 kDa
Control D-gal D-gal + GTDE GTDE Control D-gal D-gal + GTDE GTDE

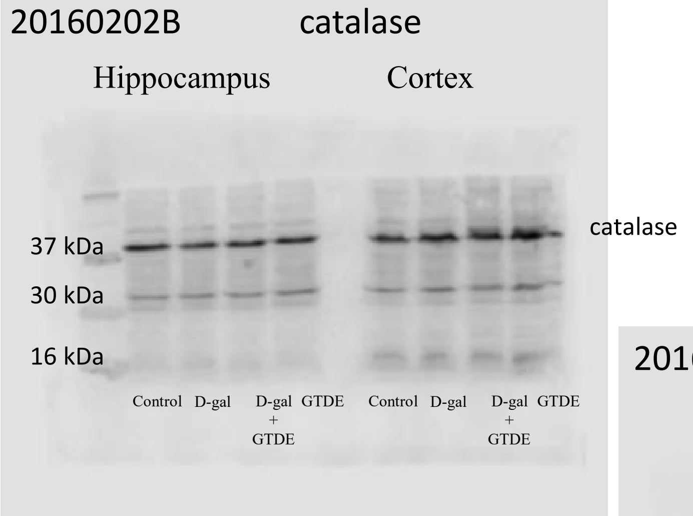
20160202B
catalase
Hippocampus
Cortex
37 kDa
30 kDa
16 kDa
catalase
Control D-gal D-gal + GTDE GTDE Control D-gal D-gal + GTDE GTDE

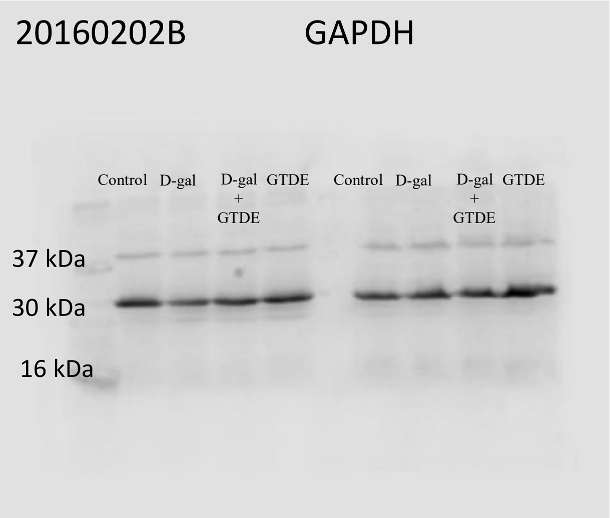
20160202B
GAPDH
Control D-gal D-gal + GTDE GTDE Control D-gal D-gal + GTDE GTDE
37 kDa
30 kDa
16 kDa

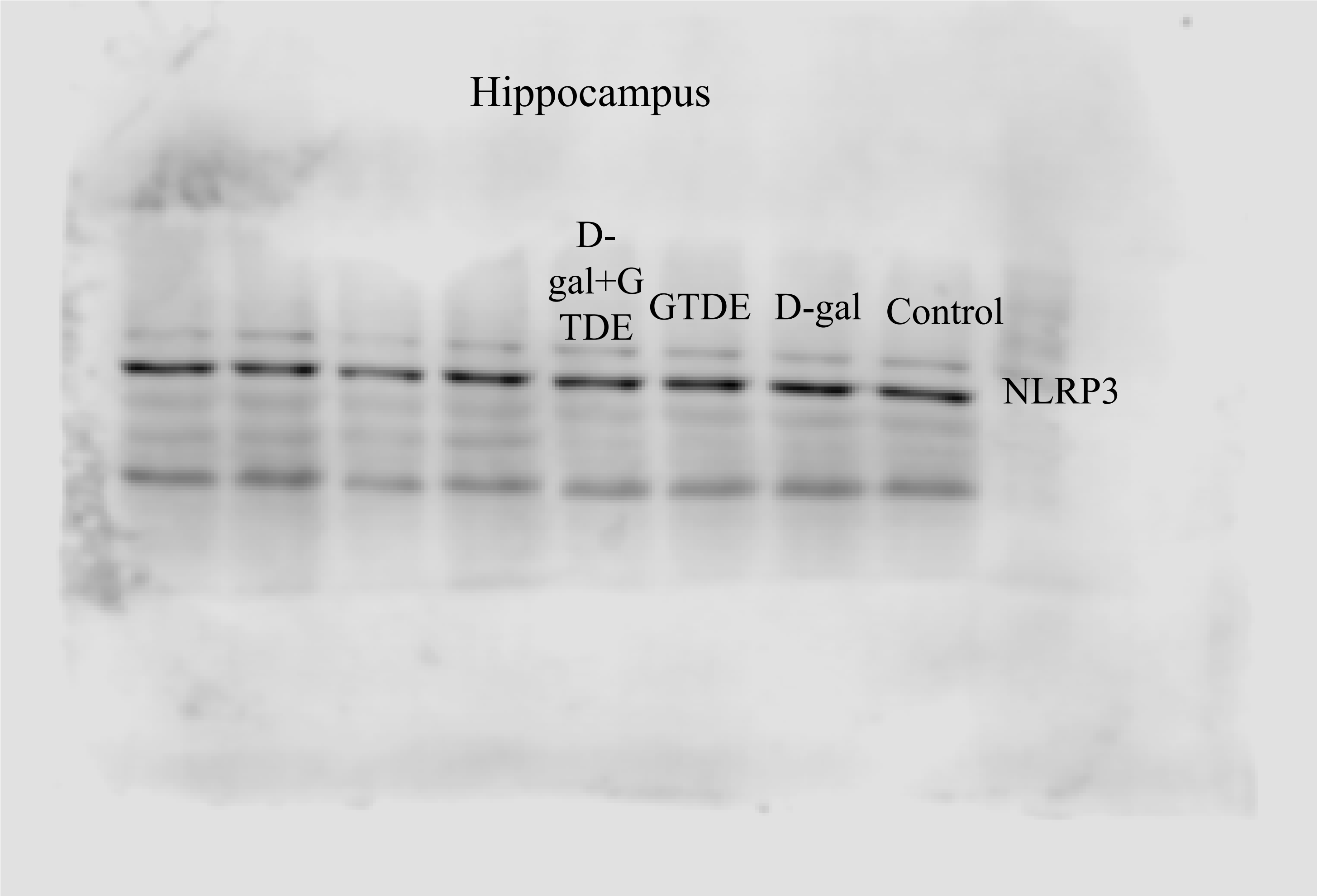
Hippocampus
D-gal+GTDE
GTDE
D-gal
Control
NLRP3

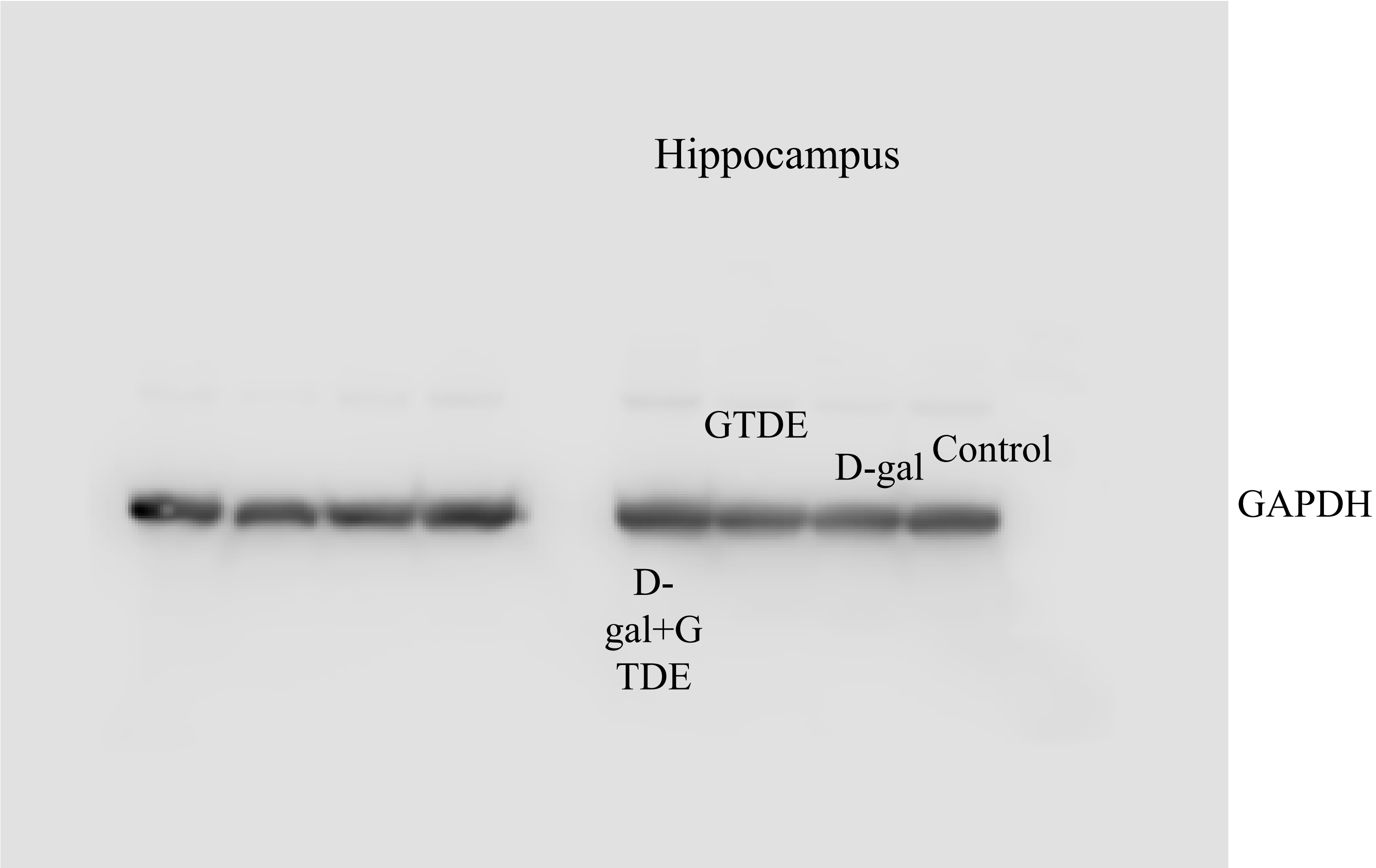
Hippocampus
GTDE
D-gal
Control
GAPDH
D-
gal+G
TDE

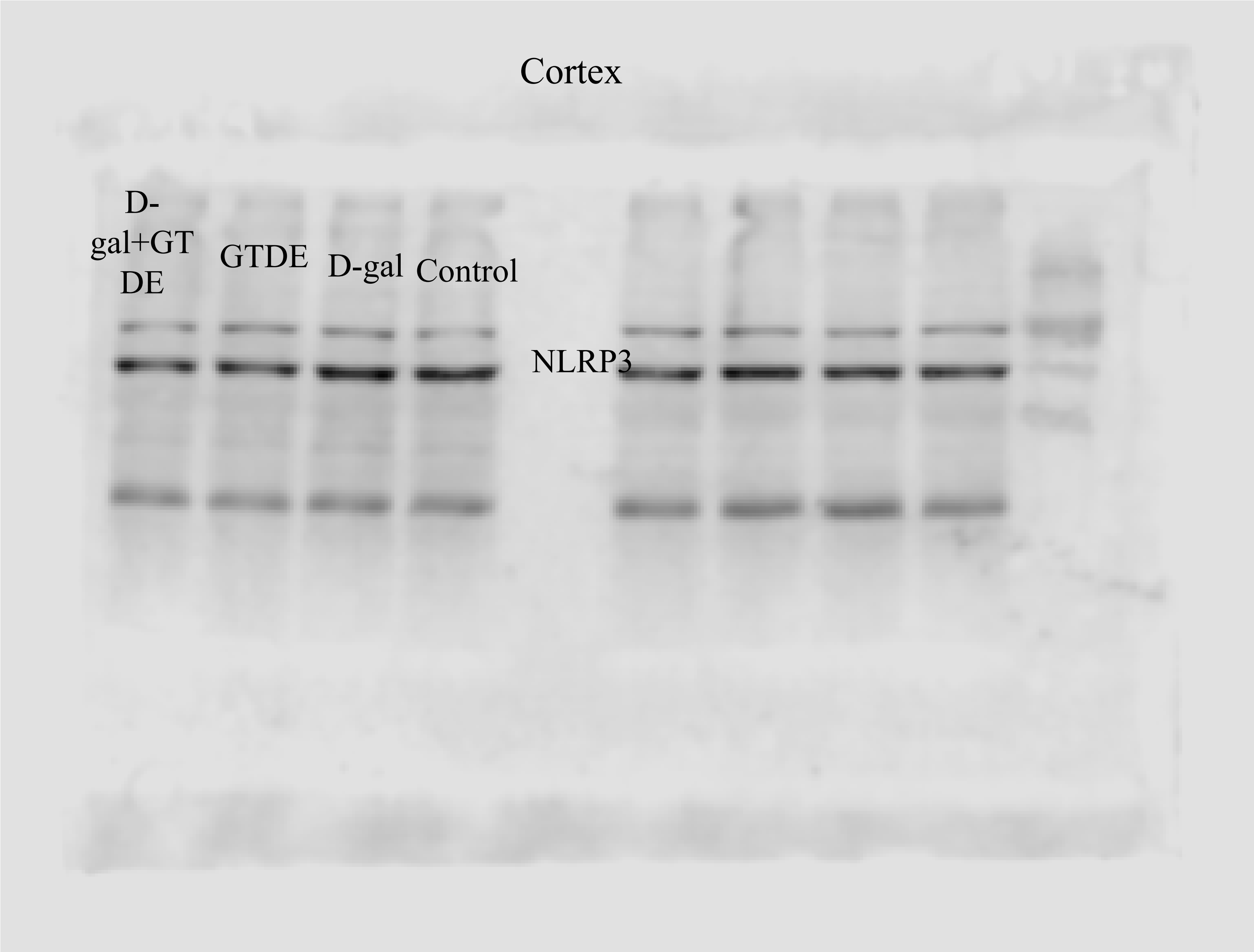
Cortex
D-gal+GTDE
GTDE
D-gal
Control
NLRP3

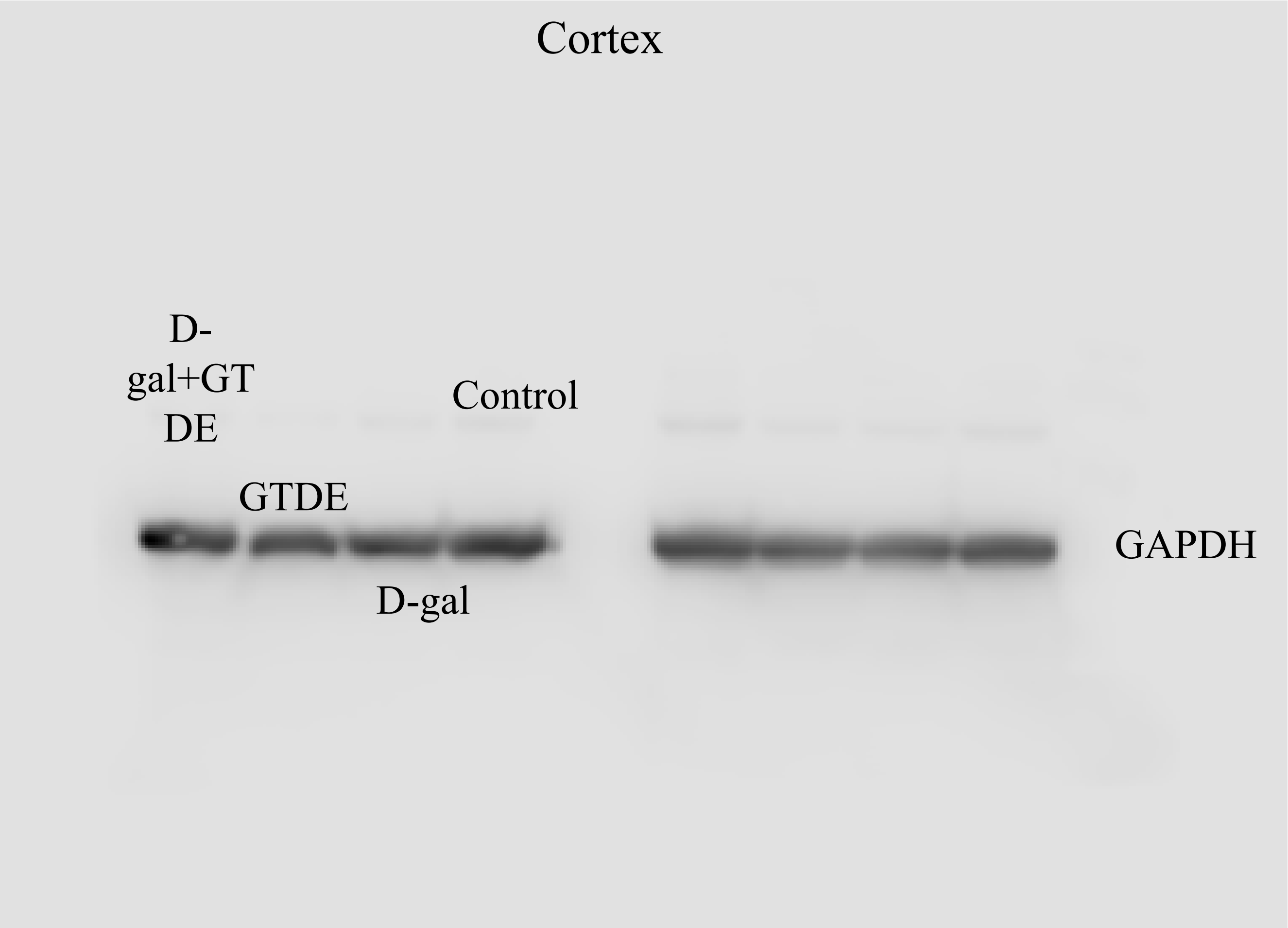
Cortex
D-gal+GTDE
GTDE
Control
D-gal
GAPDH
